# Implant-Free Breast Volume Restoration Using Abdominal Free Flaps in Nononcologic Patients: A Retrospective Cohort Study

**DOI:** 10.1093/asjof/ojag187

**Published:** 2026-10-06

**Authors:** Robert Jonathan Musmann, Mazen Hagouan, Marc Daniels, Beatrix Munder, Katrin Seidenstücker, Sonia Fertsch, Dirk Janku, Birgit Aufmesser, Nour Abu Aballah, Sogol Rajabpour, Nora Stockhausen, Annabelle Bromba, Laura Wohltat, Benedetta Vasselli, Philipp Kuhlwein, Sarina Jamchi, Andreas Wolter, Christoph Andree

## Abstract

Abdominal free flaps are established in oncologic breast reconstruction but are less frequently used for implant-free breast volume restoration in nononcologic patients. The aim of this study was to describe the indications, operative characteristics, complications, follow-up, and secondary procedures associated with abdominal free-flap breast reconstruction or augmentation in a nononcologic cohort. The authors performed a retrospective single-center cohort study of consecutive patients who underwent deep inferior epigastric perforator (DIEP) or muscle-sparing transverse rectus abdominis myocutaneous (msTRAM) flap transfer for nononcologic indications between 2004 and 2025. Patients undergoing reconstruction for oncologic breast disease were excluded. Clinical, operative, and outcome data were extracted from medical records. Surgical complications were categorized according to timing and requirement for reoperation. Elective secondary procedures were recorded separately. Fourteen patients underwent 22 abdominal free-flap reconstructions, including 19 DIEP and 3 msTRAM flaps. Primary indications were congenital breast deformity in 8 patients, primary autologous augmentation in 3, revision after aesthetic augmentation in 2, and sequelae of breast abscess in 1. Implant-associated problems were present in 8 patients. Median follow-up was 35.6 months (range, 8.2-248.5 months). No total flap loss occurred. Unplanned reoperations were required in 4 of 22 reconstructions, including 1 during the index admission and 3 after discharge. Elective secondary procedures were performed in 4 of 14 patients. In this small, heterogeneous retrospective cohort, abdominal free flaps restored breast volume without total flap loss. Unplanned reoperations and elective secondary procedures occurred during the observed follow-up period.

**Level of Evidence**: 4 (Therapeutic) 
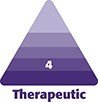

Silicone implants remain the dominant approach for breast augmentation and breast volume restoration after aesthetic breast surgery because they are widely available, reproducible, and comparatively efficient. However, implants are not a definitive solution for a relevant subgroup of patients. Capsular contracture, rupture, malposition, chronic discomfort, dissatisfaction with long-term aesthetics, and the desire for explantation or an implant-free body concept create a recurring clinical problem: how to restore or augment breast volume without reintroducing a foreign body.

Autologous tissue transfer addresses this problem conceptually by providing implant-free volume restoration with soft tissue that can age more naturally and avoids implant-related failure modes. Despite this, abdominal free flaps—especially deep inferior epigastric perforator (DIEP) flaps—are predominantly viewed as oncologic reconstructive tools in many centers rather than as an option in selected nononcologic patients. As a result, patients seeking implant-free volume restoration are often discussed in fragmented categories (eg, congenital deformity, implant revision, and explant deformity), despite overlapping reconstructive considerations across these indications.

The use of abdominal free flaps for nononcologic breast augmentation is not new. Munhoz et al described an early case of DIEP flap breast augmentation after implant-related complications, establishing the principle that perforator flaps can function as an aesthetic alternative in selected patients.^1^ Martinez and Boutros later demonstrated the feasibility of outpatient cosmetic DIEP flap augmentation combined with abdominoplasty in a small consecutive series.^2^ Zoccali et al further supported the value of free flaps in aesthetic breast surgery in selected indications, particularly in patients after massive weight loss.^3^ Additional reports have documented the role of abdominal perforator flaps in congenital deformities, such as Poland syndrome and unilateral hypoplasia.^4,5^ In tuberous breast deformity, implant-based techniques remain common, but autologous approaches including fat grafting and free flaps offer implant-free alternatives for selected patients.^6^

Despite increasing conceptual interest, the literature remains limited by small series, heterogeneous indications, mixed endpoint definitions, and inconsistent reporting of revision burden. In particular, many reports focus on flap survival and technical feasibility while underreporting the staged nature of treatment, including elective refinements that are often part of achieving the final aesthetic outcome. This makes it difficult to compare studies, to counsel patients realistically, and to position abdominal free flaps appropriately in nononcologic breast surgery.

We developed a pathway to describe a structured approach for the consideration of abdominal free-flap breast volume restoration in selected nononcologic patients, including (1) congenital deformity correction, (2) implant-related revision or implant-to-autologous conversion, and (3) primary implant-free augmentation. This pathway highlights indication-setting, comparison with alternatives, and staged counseling in an elective/revision context.

The present study reports a consecutive single-center series of abdominal-based free flaps used for nononcologic breast volume restoration between 2004 and 2025. Beyond feasibility and complication rates, we separately report flap-level safety, unplanned reoperations, elective refinements, and complication timing (index admission vs postdischarge) in order to better characterize the clinical course following these procedures.

## METHODS

We performed a retrospective single-center cohort study of consecutive abdominal-based free-flap breast reconstructions/augmentations carried out at the Department of Plastic and Aesthetic Surgery, Sana Hospital Düsseldorf-Gerresheim, between 2004 and 2025. This retrospective study was approved by the Ethics Committee of Witten/Herdecke University (reference no. S-115/2026) and was conducted in accordance with the principles of the Declaration of Helsinki. The requirement for additional study-specific informed consent for the retrospective analysis of de-identified clinical data was waived by the Ethics Committee. The focus of this study was the use of abdominal tissue transfer as a primarily nononcologic, implant-free strategy for breast volume restoration or augmentation in selected patients. Patients were eligible if they underwent abdominal free-flap transfer to the breast (DIEP or muscle-sparing transverse rectus abdominis myocutaneous [msTRAM] flap) for primarily nononcologic indications. All procedures were performed following individualized clinical evaluation and counseling regarding alternative reconstructive options.

Medical records of all included patients were reviewed using a standardized data extraction sheet. Baseline variables included age, height, weight, BMI, smoking status, comorbidities, previous abdominal surgery, and history of chemotherapy or radiotherapy. Clinical indications were categorized as congenital breast deformity (including tuberous breast deformity and Poland syndrome), primary autologous augmentation, revision surgery after aesthetic augmentation, sequelae after breast abscess, and implant-associated problems (eg, capsular contracture, systemic symptoms attributed by the patient to breast implants). Because implant-associated problems may coexist with other indication categories, these were additionally documented as overlapping indication features rather than treated as a mutually exclusive category.

Operative and perioperative variables included laterality (unilateral vs bilateral reconstruction), flap type (DIEP vs msTRAM), preexpansion before flap transfer, reconstruction after implant loss, operative time (skin-to-skin), and length of hospital stay. Where documented, perforator characteristics (single vs multiple perforators; medial vs lateral row) and inset strategy (buried flap vs skin paddle) were recorded. De-epithelialization, when performed as a planned technical step for inset/shaping, was documented but not classified as a complication. To minimize abdominal wall morbidity, DIEP flap harvest was performed using a muscle-splitting intramuscular approach, with limited opening of the anterior rectus sheath and preservation of the rectus muscle and its motor nerve branches whenever technically feasible. In msTRAM flaps, muscle sacrifice was limited to the segment required to safely incorporate the selected perforators. The anterior rectus sheath was closed primarily after pedicle harvest. Mesh reinforcement was not routinely used.

Outcomes were assessed primarily on the reconstruction/flap level, because bilateral cases contributed 2 separate flap reconstructions. Complications were stratified by timing (during index hospital stay vs after discharge) and by treatment consequence (reoperation requiring vs nonreoperation requiring). Recorded complications included hematoma, fat necrosis, partial flap loss, total flap loss, and wound-healing disorders at donor and recipient sites. Elective secondary procedures were documented separately from unplanned reoperations and included breast and abdominal contour refinements as well as symmetrizing reduction mammaplasty. Data were collected in Microsoft Excel (Microsoft Corporation, Redmond, WA) and analyzed descriptively using IBM SPSS Statistics (IBM Corp., Armonk, NY). Given the exploratory and descriptive nature of this small retrospective cohort, analyses were primarily descriptive. Continuous variables are presented as mean (range) and categorical variables as absolute numbers and percentages.

## RESULTS

Fourteen patients underwent abdominal-based autologous breast reconstruction/augmentation for primarily nononcologic indications, resulting in 22 flap reconstructions (6 unilateral cases [43%] and 8 bilateral cases [57%]; Table 1). Nineteen flaps (86.4%) were DIEP flaps, and 3 flaps (13.6%) were msTRAM flaps. The most frequent primary indication cluster was congenital breast deformity (8/14 patients, 57%), including tuberous breast deformity (5/14, 36%), Poland syndrome (2/14, 14%), and one case of Barber–Say syndrome (1/14, 7%; Table 2). Additional primary indications included primary autologous breast augmentation (3/14, 21%), revision surgery after aesthetic augmentation (2/14, 14%), and sequelae after breast abscess (1/14, 7%). Implant-associated problems were present as overlapping indication features in 8/14 patients (57%), most commonly capsular contracture (5/14, 35.7%). Systemic symptoms attributed by the patient to breast implants and persistent problems after explantation were each present in 1/14 patients (7.1%). Five patients (35.7%) explicitly sought a definitive implant-free autologous solution.

**Table 1.** Patient Characteristics (*n* = 14)

Characteristic	Value
Age, years	44.4 (24-66)
Height, cm	170.1 (157-190)
Weight, kg	76.6 (57-120)
BMI, kg/m^2^	25.8 (20.0-34.6)
Smoking	2 (14.3)
Any comorbidity	7 (50.0)
Hypertension	2 (14.3)
Asthma/COPD	2 (14.3)
Diabetes mellitus	2 (14.3)
History of renal cancer	1 (7.1)
Previous abdominal surgery	6 (42.9)
Appendectomy	3 (21.4)
Laparotomy	1 (7.1)
Cesarean section	1 (7.1)
Laparoscopy	1 (7.1)
Previous radiotherapy	0 (0)
Previous chemotherapy	0 (0)
Median follow-up, months	35.6 (8.2-248.5)

COPD, chronic obstructive pulmonary disease.

**Table 2.** Primary Indication Cluster (Mutually Exclusive; *n* = 14)

Primary indication	*n* (%)
Congenital breast deformity	8 (57.1)
Tuberous breast deformity	5 (35.7)
Poland syndrome	2 (14.3)
Barber–Say syndrome	1 (7.1)
Primary implant-free autologous augmentation	3 (21.4)
Revision after aesthetic augmentation	2 (14.3)
Sequelae after breast abscess	1 (7.1)

Mean age at surgery was 44.4 years (range, 24-66 years). Mean height was 170.1 cm (range, 157-190 cm), mean weight was 76.6 kg (range, 57-120 kg), and mean BMI was 25.8 kg/m^2^ (range, 20.0-34.6 kg/m^2^). Two patients (14.3%) were smokers. Comorbidities were documented in 7/14 patients (50.0%), including arterial hypertension in 2 (14.3%), asthma/chronic obstructive pulmonary disease in 2 (14.3%), diabetes mellitus in 2 (14.3%), and a history of renal cancer in 1 (7.1%). Previous abdominal surgery was present in 6/14 patients (42.9%), most commonly appendectomy (3/14, 21.4%); laparotomy, cesarean section, and laparoscopy were each documented in 1/14 patients (7.1% each). No patient had a history of radiotherapy or chemotherapy.

Preexpansion before flap transfer was performed in 7/14 patients (50.0%; Table 3). Reconstruction following implant loss was performed in 1/14 patients (7.1%). Mean operative time (skin-to-skin) was 377.6 minutes (range, 181-498 minutes) overall and was longer in bilateral than unilateral reconstructions (433.6 vs 303.0 minutes). Mean length of hospital stay was 7.57 days (range, 5-14 days) overall and was longer after bilateral than unilateral reconstruction (8.25 vs 6.67 days). Perforator data were available for 21/22 reconstructions (95.5%). Single-perforator flaps were used in 12/21 documented reconstructions (57.1%), predominantly based on medial-row perforators (11/12); one single-perforator flap used a lateral-row perforator. Multiple-perforator flaps were used in 9/21 documented reconstructions (42.9%). Flaps were inset without a skin paddle (buried flap) in 18/21 documented reconstructions (85.7%) and with a skin paddle in 3/21 documented reconstructions (14.3%). A 2-staged approach with second-stage de-epithelialization was performed in 3 flaps across 2 patients and was recorded as a planned technical step rather than a complication.

**Table 3.** Operative and Perioperative Variables

Variable	Value (%) (*n* = 14)
Unilateral	6 (42.9)
Bilateral	8 (57.1)
Preexpansion before flap transfer	7 (50.0)
Reconstruction following implant loss	1 (7.1)
Operative time (skin-to-skin), min	377.6 (181-498)
Unilateral cases, mean min	303
Bilateral cases, mean min	433.6
Length of stay, days	7.57 (5-14)
Unilateral cases, mean days	6.67
Bilateral cases, mean days	8.25

Variable	Value (%) (*n* = 22)
Total reconstructions/flaps	22
DIEP flaps	19 (86.4)
msTRAM flaps	3 (13.6)
Perforator data available	21/22 (95.5)
Single-perforator flaps	12/21 (57.1)
Medial row (among single perforator)	11/12 (91.7)
Lateral row (among single perforator)	1/12 (8.3)
Multiple-perforator flaps	9/21 (42.9)
Inset strategy available	21/22 (95.5)
Buried flap (no skin paddle)	18/21 (85.7)
Skin paddle used	3/21 (14.3)
Second-stage de-epithelialization	3/22 (13.6)

DIEP, deep inferior epigastric perforator; msTRAM, muscle-sparing transverse rectus abdominis myocutaneous.

No total flap loss occurred (Table 4). Major surgical complications (reoperation requiring) occurred in 4/22 reconstructions (18.2%), including 1/22 (4.55%) during the index hospital stay and 3/22 (13.64%) after discharge. The in-hospital reoperation-requiring event was a hematoma (1/22, 4.55%). Postdischarge reoperation-requiring complications included abdominal donor-site wound-healing problems in 2/22 reconstructions (9.09%), fat necrosis in 1/22 (4.55%) resulting in partial flap loss in the same case. No breast wound-healing disorders were documented. Minor surgical complications (nonreoperation requiring) occurred in 0/22 reconstructions. Medical complications occurred in 2/14 patients (14.3%) during hospitalization and were not observed after discharge. This included 1 case of a pulmonary embolism and 1 exacerbation in a patient with obstructive lung disease. Elective secondary procedures were performed in 4/14 patients (28.6%; Table 5). These included symmetrizing reduction mammaplasty (2 procedures), abdominal scar revision (2 procedures), and breast scar revision and contouring (1 procedure). The median follow-up was 35.6 months (range, 8.2-248.5 months). No abdominal bulge or hernia was documented during follow-up.

**Table 4.** Major Surgical and Nonsurgical Complications

Complication	Index admission: total (%)	Postdischarge: total (%)
Surgical (*n* = 22 flaps)		
Hematoma	1 (4.55)	0
Fat necrosis	0	1 (4.55)
Partial flap loss	0	1 (4.55)
Total flap loss	0	0
Donor-site wound-healing disorder	0	2 (9.09)
Recipient-site wound-healing disorder	0	0
Nonsurgical (*n* = 14 patients)		
Pulmonary embolism	1 (7.14)	0
COPD flare	1 (7.14)	0

Fat necrosis and partial flap loss occurred in the same reconstruction.

COPD, chronic obstructive pulmonary disease.

**Table 5.** Elective Secondary Procedures (Patient-level; *n* = 14)

Variable	Value (%)
Patients with ≥1 elective secondary procedure	4 (28.6)
Total elective secondary procedures	5
Symmetrizing reduction mammaplasty, procedures	2
Abdominal scar correction, procedures	2
Breast scar and contour correction, procedures	1

## DISCUSSION

In our consecutive cohort, abdominal free tissue transfer was feasible across 3 clinically relevant indication clusters—congenital deformity correction, primary implant-free augmentation, and implant-related revision/conversion—with no total flap loss. Beyond demonstrating technical feasibility, our series highlights the heterogeneity of nononcologic indications for abdominal free-flap breast reconstruction/augmentation and the importance of detailed reporting of complications, reoperations, and elective secondary procedures beyond flap survival alone. At the same time, the observed reoperation-requiring complications and secondary procedures emphasize that these procedures represent complex microsurgical reconstructions requiring careful patient selection and detailed preoperative counseling.

This distinction matters because outcome reporting in the current literature is often heterogeneous and difficult to interpret clinically. Published series frequently combine different indications and focus primarily on flap survival while providing limited granularity regarding timing of complications, donor-site morbidity, and the role of planned aesthetic refinements. In the nononcologic setting, this can be misleading: a patient may have an excellent flap outcome but still require staged symmetry or contour procedures as part of the intended treatment plan. We therefore deliberately separated flap-level safety, unplanned reoperations, elective secondary procedures, and complication timing (during index admission vs after discharge). Standardized reporting of these outcomes may therefore improve comparability between studies and provide a more realistic representation of the reconstructive pathway experienced by patients.

Our findings are broadly consistent with previous reports demonstrating the feasibility of abdominal free flaps in aesthetic and revision breast surgery. Martinez and Boutros reported outpatient cosmetic DIEP augmentation in 11 patients without flap loss, whereas Zoccali et al described favorable outcomes in a selected cohort undergoing autologous tissue transfer for cosmetic indications.^2,3^ Musmarra et al reported bilateral DIEP flap reshaping in postbariatric patients with meaningful patient-reported improvement and a low flap loss rate.^7^ Compared with these reports, our series adds a broader real-world nononcologic spectrum and places greater emphasis on the practical reporting of the overall treatment pathway rather than on technical innovation alone. The absence of total flap loss in our cohort supports the technical feasibility of this strategy within a specialized microsurgical setting, whereas the observed revision burden underscores the importance of expectation management. The observed reoperations demonstrate the relevant treatment burden associated with these procedures.

An important clinical observation in this cohort is that abdominal free-flap reconstruction was used across several nononcologic indication clusters that are often discussed separately despite overlapping reconstructive considerations. These included implant-related revision or conversion, congenital breast deformities, and primary implant-free augmentation. Although the underlying clinical problems differed, all groups required an individualized assessment of the volume and quality of the existing breast envelope, the likelihood of achieving the treatment objective with less invasive techniques, the availability of abdominal donor tissue, and the patient's willingness to accept the morbidity and recovery associated with microsurgical reconstruction.

In our cohort, implant-related revision/conversion represented a substantial proportion of cases, with capsular contracture being the dominant implant-associated problem. This mirrors larger oncologic conversion series, where poor cosmesis and capsular contracture are among the leading reasons for tertiary autologous reconstruction after implants.^8-12^ For these patients, the clinical value of abdominal free flaps lies not only in volume restoration but also in offering a definitive implant-free endpoint, which was achieved in the observed follow-up period. This is particularly relevant in patients who have already undergone multiple implant procedures and prioritize avoidance of further implant-related failure modes (Figure 1). However, the complexity of salvage surgery—including scarring and vessel-related challenges—must be addressed transparently during counseling.^9^ Larger tertiary reconstruction series have demonstrated that DIEP flap conversion after failed implant-based reconstruction is feasible, with low flap loss rates but a relevant burden of additional procedures, consistent with the pathway observed in our cohort.^8-10^

**Figure 1.**
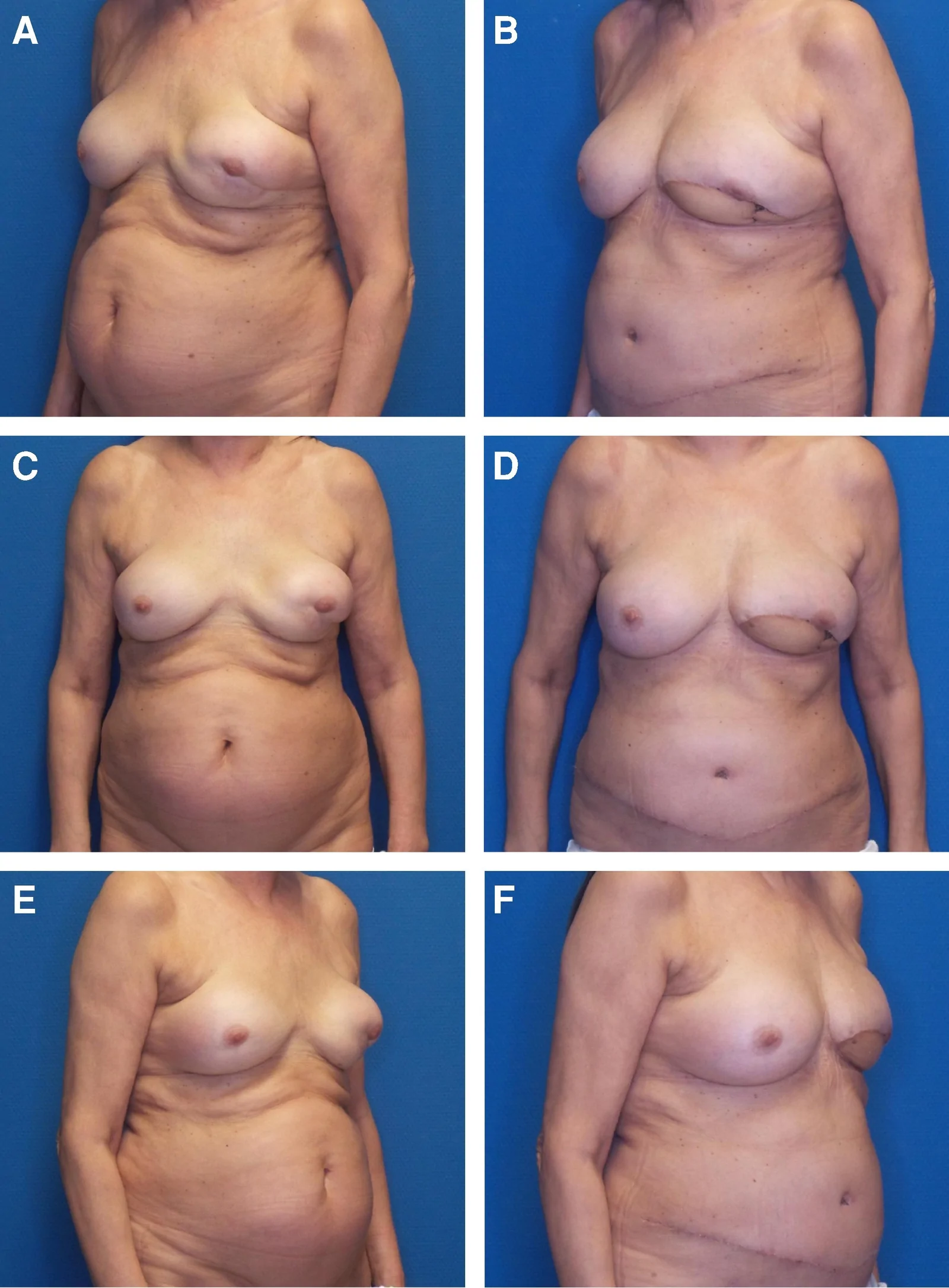
Implant-related complication after aesthetic augmentation. Female patient: 66 years; BMI 21.8 kg/m^2^. Indication: implant-exit because of capsular contracture (Baker grade IV) 20 years following aesthetic augmentation. (A) Preoperative oblique left view and (B) postoperative oblique left view, 6 months after bilateral DIEP transfer. (C) Preoperative frontal view and (D) postoperative frontal view, 6 months after bilateral DIEP transfer. (E) Preoperative oblique right view and (F) postoperative oblique right view, 6 months after bilateral DIEP transfer. Previous implants were manufacturer Allergan, volume 180 mL, placed in the subpectoral plane through inframammary approach in year 2005. DIEP, deep inferior epigastric perforator.

The congenital deformity cluster deserves separate consideration because it illustrates the limitations of a purely implant-based reconstructive approach (Figures 2, 3). In tuberous breast deformity and Poland syndrome, the primary problem is often not only volume deficiency but also shape, base constriction, asymmetry, and soft-tissue quality. Implant-based correction may improve volume while leaving structural deformity incompletely addressed or creating new revision cycles (Figure 4). Autologous tissue transfer offers a different reconstructive logic: soft-tissue replacement and reshaping without foreign material. Previous reports support the use of perforator flaps in selected congenital cases, with favorable long-term outcomes and acceptable revision rates.^4-6,13-16^ This does not make abdominal free flaps a first-line option for all congenital deformities, but it supports their role in selected patients—particularly those with previous failed implant-based treatment, complex asymmetry, or a strong preference for implant-free reconstruction.

**Figure 2.**
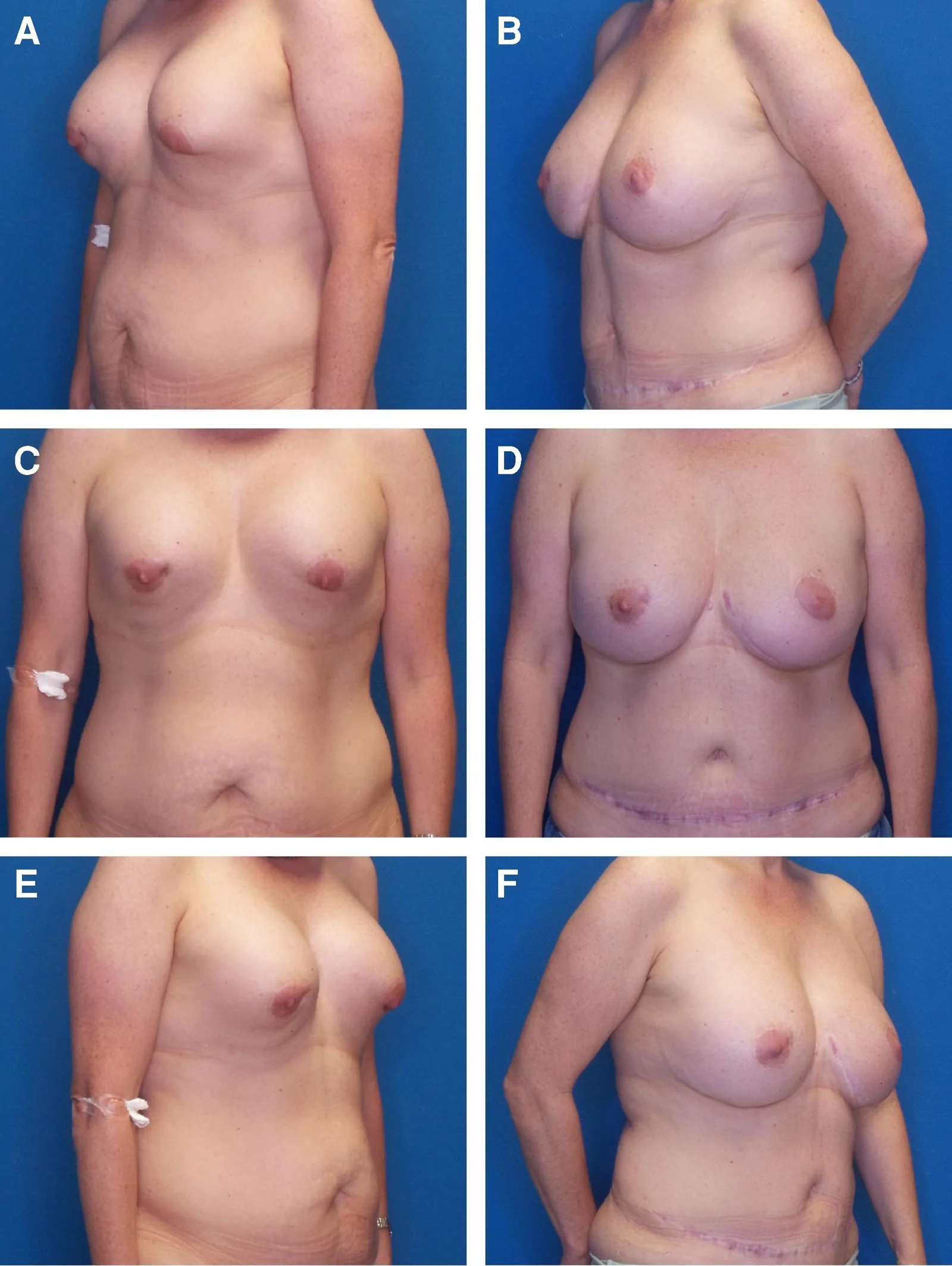
Combined congenital deformity and implant-related complication. Female patient: 47 years; BMI 23.8 kg/m^2^. Indication: implant-exit because of capsular contracture (Baker grade IV) 12 years following implant reconstruction because of tuberous breast deformity. (A) Preoperative oblique left view and (B) postoperative oblique left view, 36 months after bilateral DIEP transfer. (C) Preoperative frontal view and (D) postoperative frontal view, 36 months after bilateral DIEP transfer. (E) Preoperative oblique right view and (F) postoperative oblique right view, 36 months after bilateral DIEP transfer. Previous implant manufacturer and volume are unknown. DIEP, deep inferior epigastric perforator.

**Figure 3.**
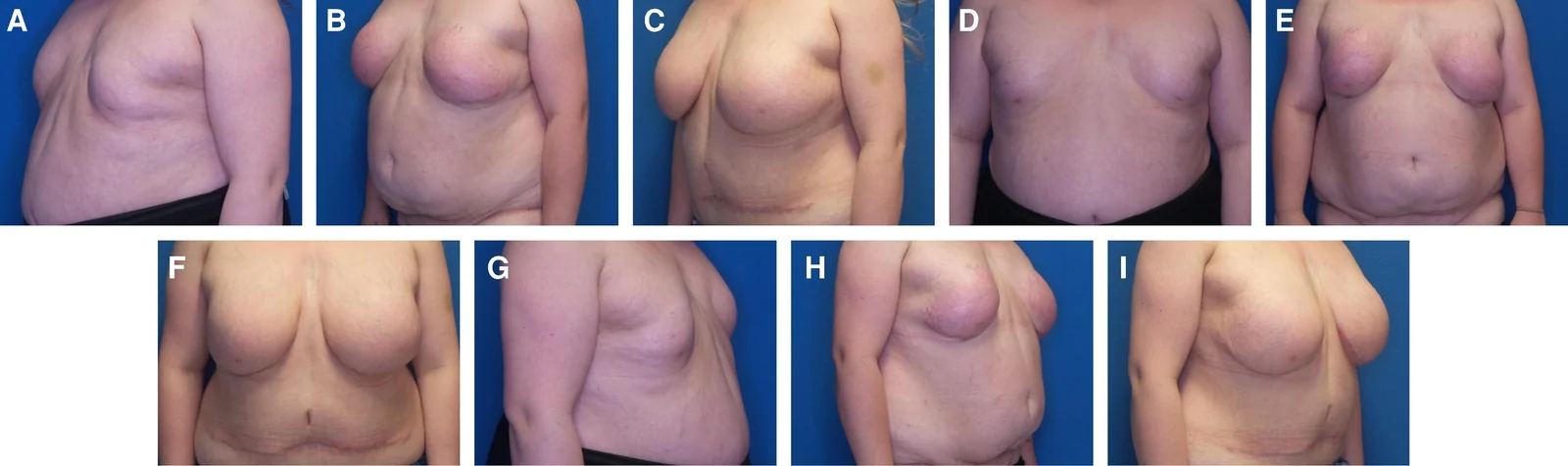
Breast aplasia in the context of Barber–Say syndrome. Female patient: 25 years; BMI 34.0 kg/m^2^. Indication: Breast aplasia in the context of Barber–Say syndrome. Images A, D, and G show the preoperative appearance (A: oblique left, D: frontal, G: oblique right). Images B, E, and H show the appearance after tissue expansion (16 weeks after expander insertion; initial fill 300 mL; final fill 500 mL; 6 weeks after final fill) (B: oblique left, E: frontal, H: oblique right). Images C, F, and I show the postoperative result 7 months after bilateral DIEP transfer and operative abdomen scar revision because of wound dehiscence (C: oblique left, F: frontal, I: oblique right). DIEP, deep inferior epigastric perforator.

**Figure 4.**
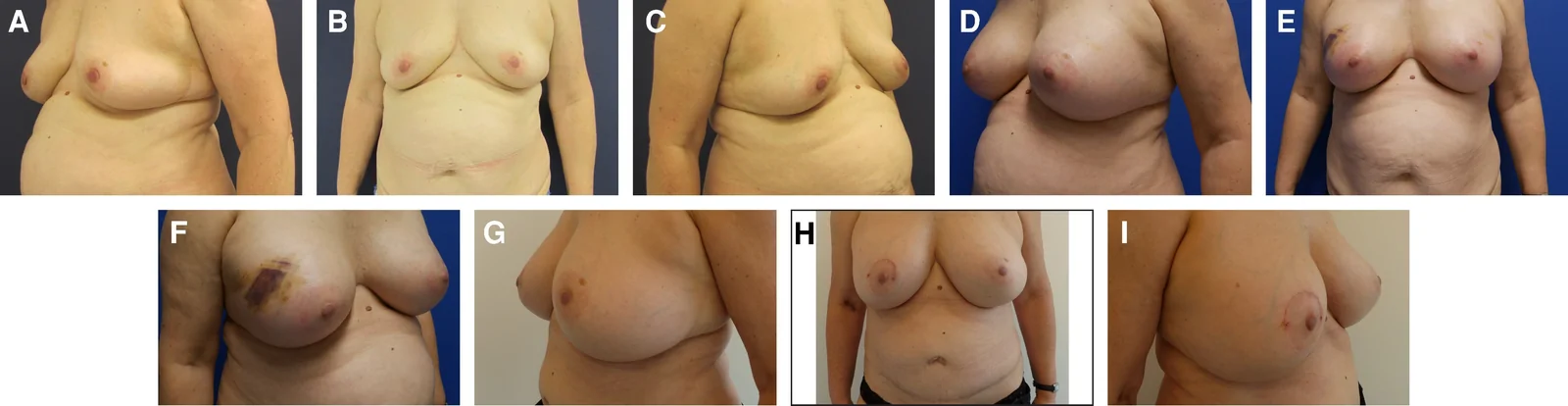
Primary implant-free breast augmentation using staged abdominal free-flap reconstruction. Female patient: 56 years; BMI 23.1 kg/m^2^. Indication: Primary implant-free breast augmentation. Images A, D, and G show the preoperative appearance (A: oblique left, D: frontal, G: oblique right). Images B, E, and H show the appearance after tissue expansion (12 weeks after expander insertion; initial fill 200 mL; final fill 350 mL; 5 weeks after final fill) (B: oblique left, E: frontal, H: oblique right). Images G-I show the postoperative result 15 months after bilateral DIEP transfer (G: oblique left, H: frontal, I: oblique right). DIEP, deep inferior epigastric perforator.

Primary autologous breast augmentation represents the most controversial indication within this spectrum. It may constitute a potential option for a highly selected subgroup of patients who strongly prioritize autologous reconstruction and implant avoidance despite the increased surgical complexity, scarring, donor-site morbidity, and possibility of staged refinements associated with microsurgical tissue transfer (Figure 4).^2^ The 3 cases of primary autologous augmentation in this historical cohort should not be interpreted as support for the routine use of abdominal free flaps in patients with normal breast anatomy and isolated cosmetic hypomastia. Implant-based augmentation was technically possible in these patients but did not fulfill the complete treatment objective because permanent foreign material was explicitly declined. In addition, all 3 patients had substantial abdominal skin and soft-tissue excess and independently desired abdominal contouring, allowing tissue that would otherwise have been discarded to be used for breast volume restoration. All 3 patients who underwent primary implant-free augmentation underwent an individualized and documented ethical and clinical assessment. Conventional implant augmentation and staged fat grafting were discussed as alternatives. Each patient understood that abdominal free-flap augmentation was not equivalent to routine cosmetic breast augmentation and accepted the higher operative morbidity, potential flap loss, risk of unplanned reoperation, substantial abdominal and breast scarring, prolonged recovery, possible staged refinements, and the financial implications of nonmedically necessary surgery. None of these patients were recruited through procedure-specific marketing.

Staged fat grafting represents a less invasive autologous alternative in this setting and should be considered whenever the required volume is limited and the breast envelope is suitable. However, it may require multiple procedures, offers variable volume retention, and would not have independently addressed the abdominal skin excess present in these patients. The comparison with fat grafting is therefore not limited to achievable breast volume alone but also includes the number of anticipated procedures, donor-site objectives, predictability of volume restoration, and the overall treatment burden.

Because patient selection and expectation management are central determinants of perceived success in this setting, a structured clinical evaluation process may help determine when abdominal free-flap reconstruction can reasonably be considered and when alternative strategies are more appropriate (Figure 5). Abdominal free tissue transfer should not be regarded as a first-line alternative to conventional breast augmentation or routine implant revision. Its consideration requires a stepwise assessment of the reconstructive problem, the available less invasive alternatives, patient-specific risk factors, the donor-site situation, and the expected benefit relative to the morbidity of microsurgical reconstruction.

**Figure 5.**
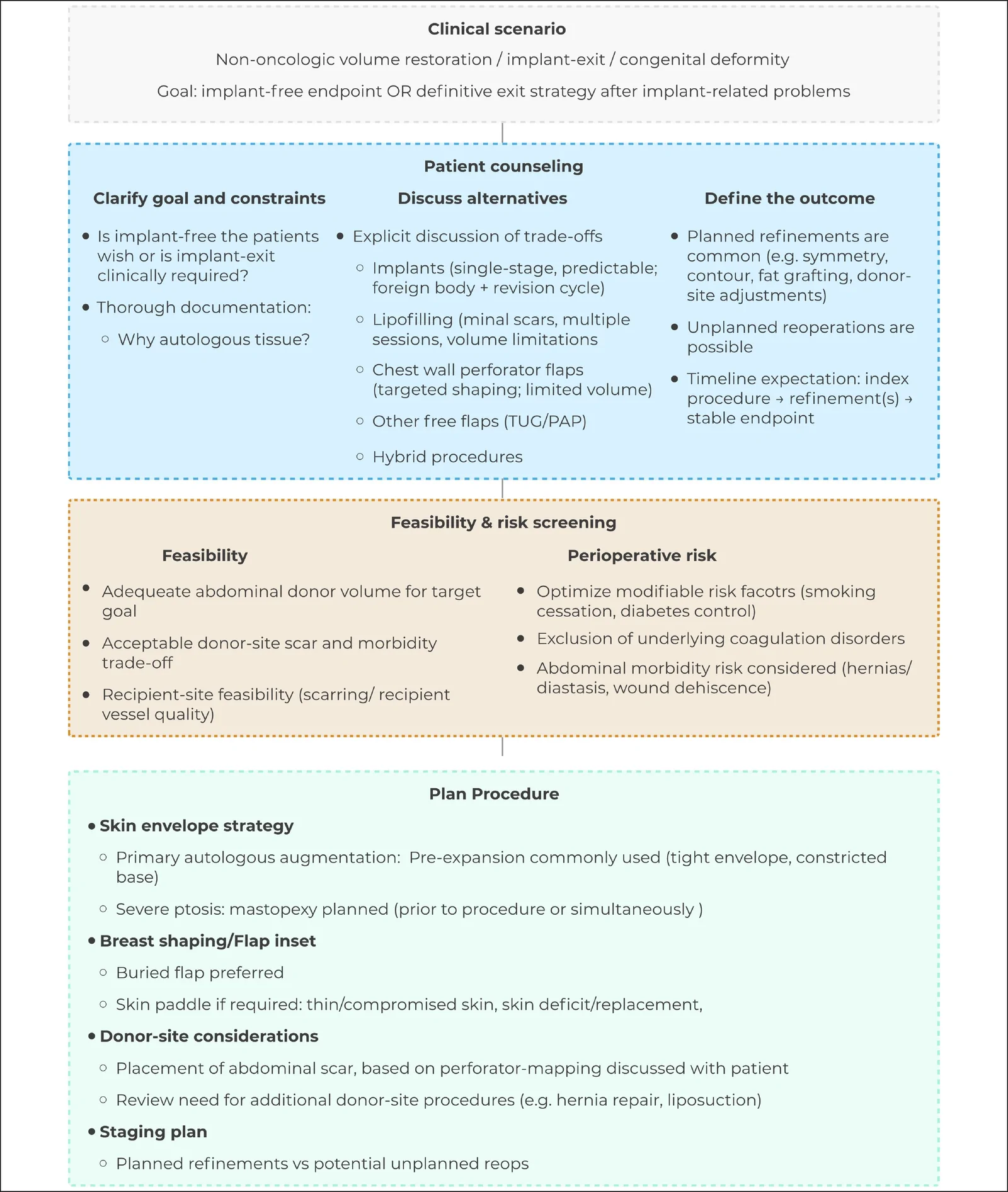
Clinical considerations for abdominal free-flap reconstruction in selected nononcologic patients. Schematic illustration of clinical considerations relevant to abdominal free-flap breast reconstruction in selected nononcologic patients. The figure summarizes factors that may be considered during patient counseling and preoperative evaluation, including reconstructive goals, alternative treatment options, donor-site considerations, surgical complexity, and patient expectations. The figure is intended as a descriptive conceptual illustration rather than a validated clinical algorithm.

Implant revision or replacement remains appropriate for patients who accept continued implant surveillance and who do not have recurrent implant-related complications or severely compromised soft tissues. Staged fat grafting may be preferable when the required volume is limited, the breast envelope is suitable, and the patient prioritizes lower operative morbidity despite the potential need for multiple procedures and variable graft retention. In contrast, an abdominal free flap may be considered in selected patients with complex congenital deformity, substantial volume and soft-tissue deficiency, or recurrent implant-related problems when implants or staged fat grafting are unlikely to achieve the reconstructive objective or are not acceptable to the patient.

However, a preference for implant avoidance and the mere availability of abdominal donor tissue are not sufficient indications for microsurgical breast augmentation. The rationale must be exceptional, individualized, and clearly documented. The assessment should include the availability of sufficient donor tissue, previous abdominal surgery, comorbidities, smoking status, thromboembolic risk, anticipated donor-site morbidity, and the patient's acceptance of prolonged recovery, abdominal and breast scarring, potential flap loss, unplanned reoperation, and staged refinements. The expected reconstructive benefit must outweigh these burdens. Patients with unrealistic expectations, those primarily seeking routine cosmetic augmentation, or those prioritizing minimal surgical morbidity and rapid recovery should not undergo this procedure.

These interventions should also be limited to established microsurgical centers with appropriate experience in patient selection, free tissue transfer, perioperative monitoring, management of flap-related complications, and secondary aesthetic refinement. The structured pathway presented in Figure 5 is not intended to represent a validated prediction model or a rigid decision algorithm. Rather, it should be understood as a conceptual clinical framework derived from retrospective real-world practice. Its purpose is to structure counseling, procedural planning, and documentation across a heterogeneous indication spectrum and to reduce discrepancies between expected and achievable outcomes.

This study has several limitations. Its retrospective design, small sample size, and single-center setting limit generalizability and preclude robust comparative inference. The indication spectrum was intentionally heterogeneous and should be interpreted as descriptive real-world pathway data rather than a uniform disease entity. Documentation quality may have affected the completeness of perforator-level details and postdischarge event capture. In addition, patient-reported outcome measures were not systematically available, limiting conclusions regarding patient-reported satisfaction and quality of life. Future multicenter studies should use standardized reporting of flap-level safety, unplanned reoperations, elective refinements, and patient-reported outcome measures to assess not only microsurgical success but also pathway completion and patient-centered benefit. Furthermore, the relatively small sample size reflects the rarity and heterogeneity of this indication spectrum rather than a lack of clinical relevance, and future multicenter studies will be required to further validate these observations.

## CONCLUSIONS

Abdominal-based free-flap reconstruction and augmentation were used in selected nononcologic patients, including congenital breast deformities and implant-related revision cases. In this retrospective single-center cohort, no total flap loss occurred, although reoperation-requiring complications and elective secondary procedures were observed.
